# Toxic Leadership in Hospital Nursing and Its Psychological Impact on Staff Well‐Being: A Scoping Review

**DOI:** 10.1155/jonm/8025182

**Published:** 2026-07-30

**Authors:** Alix Cooke, Gideon de Jong, Joanne Hilder

**Affiliations:** ^1^ Queensland Health, Brisbane, Australia, health.qld.gov.au; ^2^ Faculty of Health, Southern Cross University, Lismore, Queensland, Australia, health.qld.gov.au

**Keywords:** dark leadership, destructive leadership and nursing management, psychological impacts, toxic leadership, transformational leadership

## Abstract

**Aim:**

This scoping review examines the psychological impacts on staff resulting from toxic leadership in healthcare, specifically in hospital nursing, and offers recommendations to mitigate and manage this dark side of leadership.

**Background:**

Toxic leadership can have serious psychological effects on hospital staff, negatively impacting their health. Beyond individual well‐being, it can also undermine hospitals’ efforts to increase risks to patient safety and ultimately contribute to poorer patient outcomes.

**Method:**

Following PRISMA guidelines, a systematic search for both quantitative and qualitative evidence across four databases yielded 10 quantitative research articles, examining the experiences of 4850 healthcare professionals (3499 hospital nurses identified across 9 articles) worldwide with toxic leadership.

**Results:**

Five key themes were identified. Themes underlined that toxic leadership has a profoundly negative impact on nurses’ psychological well‐being and emotional functioning, with a final theme identifying strategies for mitigating its effects.

**Conclusion:**

The findings confirm that psychological impacts for nurses encountering toxic leadership include anxiety, burnout, depression, emotional exhaustion, low motivation, malaise, negative affectivity, stress and withdrawal. Adopting the transformational leadership style in healthcare should be the gold standard, coupled with 360‐degree appraisals. Future qualitative research is required to provide a more in‐depth understanding of the psychological impacts of toxic leadership. Its associated costs can be substantial and warrant further exploration.

**Implications for Nursing Management:**

Strengthening nurses’ awareness of toxic leadership, how to manage or escalate toxic leadership styles, and understanding its psychological impacts are essential tools for nursing management. Ensuring a supportive culture for reporting and recovery for those impacted by toxic leadership is vital, ultimately improving healthcare delivery, staff well‐being and hospital performance.

## 1. Introduction

Effective leadership is essential for healthcare staff to deliver high‐quality services that achieve optimal hospital performance and ensure positive patient safety outcomes [1]. Healthcare organisations can flourish or fail based on leadership [2]. A positive workplace culture, driven by strong, empathetic leadership, can elevate performance, staff well‐being and patient outcomes. Under poor leadership, even the most well‐resourced healthcare organisations risk dysfunction, burnout and ultimately failure [2].

Poor leadership, also referred to as toxic leadership, is an umbrella term for negative leadership styles, including destructive, abusive, narcissistic and despotic [3]. Toxic leadership is often described as the dark side of leadership and has garnered significant attention over the last decade, particularly within the healthcare sector [4]. It can reduce staff retention and negatively impact staff health [3]. This, in turn, can decrease a health professional’s functioning, negatively affect health service delivery and performance, reduce positive patient outcomes and exacerbate a poor workplace culture [3].

Continuous toxic leadership can cause various health issues for staff, including physical, psychological and emotional harm [5]. Webster et al. [5] argue that healthcare organisations should be responsible for preventing staff from being exposed to toxic or destructive behaviours. If the organisation’s administration fails to prevent staff from experiencing toxic leadership, it can have psychological impacts on senior management, resulting in repercussions for middle management and cascading downwards throughout the organisation, ultimately leading to chaos [6]. A recent systematic review by Labrague [4] provides moderate evidence suggesting that working under a leader who exhibits toxic leadership may have adverse consequences for the nursing workforce, including increased turnover intention and reduced organisational performance. Toxic leadership can therefore result in productivity loss, reputational damage, and legal costs. For example, it is estimated that employers in the United States of America (USA) incur expenditures of 23.8 billion dollars annually due to poor leadership [7].

Wilson [8] conducted quantitative research on psychological safety and toxic leadership within a federal government department in the USA with a healthcare‐related focus. Results demonstrated that as toxic leadership behaviours increased, psychological safety decreased [8]. In addition, Valentine et al. [9] provide insight into work poly‐victimisation, which refers to the aggregate load of exposure that a health professional experiences from multiple types of victimisation. Valentine et al. [9] discuss the decrease in the psychological well‐being of employees due to poly‐victimisation.

According to Durrah et al. [10] and Trépanier et al. [11], prior research has highlighted the positive effects of leaders’ behaviours on healthcare employees; however, less attention has been given to the impacts of poor leadership styles in healthcare in general and hospital nursing specifically. The studies of Durrah et al. and Trépanier et al., both published in the past decade, suggest that the impacts of toxic leadership appear to be widespread and can cause numerous negative outcomes, both intrinsically and extrinsically. To the best of our knowledge, no attempt has yet been made to systematically search for the evidence and reveal overarching patterns that can be discovered across the various studies. This prompted us to conduct a scoping review in order to close this gap and identify new areas for further exploration.

## 2. Methods

Primary research was reviewed to investigate the potential adverse psychological outcomes of exposure to toxic leadership in hospital nursing. According to Munn et al. [12], the most favourable research question format for this type of review is the population, exposure and outcome (PEO). This led to the following question: *What are the psychological impacts (O) of toxic leadership (E) on healthcare professionals and hospital nurses (P)?*

The review typology for this research topic is experiential, which focuses on human experiences and phenomena [12]. Although experiential reviews are commonly based on qualitative data, certain types of quantitative data can also be considered. Due to a lack of qualitative data, the aim of this scoping review has been addressed using quantitative data only.

The American Psychological Association (APA) PsycInfo, Cumulative Index of Nursing and Allied Health Literature (CINAHL), Medline and Scopus electronic databases were accessed to conduct systematic literature searches. These databases were chosen because they are relevant to the healthcare sector. APA PsycInfo was a particularly significant database to access for researching the psychological impacts of toxic leadership. CINAHL focuses specifically on nursing and allied health. Medline is the database for the National Library of Medicine. Scopus is a comprehensive database for academic journals across multiple industries. Key search terms were selected and combined with Boolean operators: psychological impacts of toxic leadership AND management OR senior management OR executive across all databases, and with Scopus included NOT military OR education OR *COVID-19*. APA PsycInfo, CINAHL and Medline had the same key search fields and design. Due to its design and functionality, Scopus required additional delimiters and key search fields.

The scoping review originally focused on the psychological impacts of toxic leadership across the entire multidisciplinary healthcare workforce and did not specifically target the nursing profession. Due to the overwhelming response from studies exploring the experiences of nurses, we decided to limit the scope to hospital nursing.

Secondary and grey literature sources were excluded from this review, as the authors chose to focus on robust evidence published in reputable peer‐reviewed journals. This has led to the omission of reports from universities (including PhD dissertations) and other research institutions that meet rigorous research standards and could effectively help address the aim of our scoping review.

The search strategy, including the databases, key search terms, and the number of articles found using this method, is shown in Table 1. The search was undertaken in December 2025. It was limited to articles published between 2014 and 2025 to ensure that the evidence and literature reflected a contemporary definition of psychological trauma.

**TABLE 1:** Databases and search criteria used to identify literature for review.

Database	Search terms	No. articles
APA PsycInfo	Psychological Impacts OR Toxic Leadership OR Destructive Leadership	*N* = 23
AND	Senior Management OR Management OR Executive	
OR	Toxic Leadership	
Limiters:	2014–2024, English and peer‐reviewed	

CINAHL	Psychological Impacts OR Toxic Leadership OR Destructive Leadership	*N* = 33
AND	Senior Management OR Management OR Executive	
OR	Toxic Leadership	
Limiters:	2014–2024, English and peer‐reviewed	

Medline	Psychological Impacts OR Toxic Leadership OR Destructive Leadership	*N* = 33
AND	Senior Management OR Management OR Executive	
OR	Toxic Leadership	
Limiters:	2014–2024, English and peer‐reviewed	

Scopus	Psychological Impacts OR Toxic Leadership OR Destructive Leadership	*N* = 12
AND	Senior Management OR Management OR Executive	
OR	Toxic Leadership	
NOT	Military or Education, or COVID‐19	
Limiters:	2014–2024, English, articles; nursing, medical and psychology	

Total records identified after database searching		*N* = 101

Total records after duplicates removed		*N* = 69

The following inclusion criteria were applied during article selection:

• Publication date from 2014 to 2025
• The title and aim/s were relevant to the topic
• The title and abstract discussed toxic leadership and/or psychological impacts in relation to health professionals in general and hospital nurses specifically
• Full text, peer‐reviewed journal articles in the English language only
• Qualitative, quantitative and mixed‐method study designs

These criteria ensured that appropriate literature was reviewed and the research question was adequately addressed.

This literature search method yielded 101 articles. Once duplicates (*n* = 32 articles) were removed, the titles and abstracts of 69 articles were screened for inclusion in the review. A further 51 articles were excluded because the majority focused on aspects of toxic leadership other than hospital‐related issues, were unrelated to hospitals, or were literature reviews (i.e., secondary evidence). For example, Abbas Khan [13] conducted an online questionnaire on toxic leadership within the virtual work setting during the COVID‐19 pandemic. Whilst this was an informative article, it was not within the healthcare sector. Since this study was specifically focused on COVID‐19, a turbulent and pressured period, including staff navigating personal and professional stress, we felt that even informative primary studies on leadership under pressure, including those related to COVID‐19, would be too specific. The exclusion criteria were consequently expanded to cover articles related to COVID‐19. Chua and Murray [14] conducted primary research on how toxic leaders were perceived from a gender‐based perspective. Although a thought‐provoking article, it did not address healthcare‐related issues or discuss the psychological impacts.

The remaining 18 full‐text articles were screened for inclusion in this review. A further 11 articles were excluded for the following reasons:

• Alsadaan and Alqahtani [15] discussed toxic leadership in the emergency department of a hospital. After reading the full‐text article, it was deemed too specific because different specialties have different requirements, demands and functions. The specificity could introduce bias and was not representative of the broader context of hospital nursing.
• The abstracts and titles from Farghaly Abdelaliem and Abou Zeid [16], Guo et al. [17], Koç et al. [18], Labrague [4], Ofei et al. [19], She et al. [20], Shipl et al. [21] and Sun et al. [22] all examined toxic leadership. However, the full‐text articles did not include specific research regarding psychological impacts and instead focused on the effects, predictors and perceptions of toxic leadership.
• Bulutlar et al. [23] focused on neoliberalism within healthcare and the impact of managerialism on toxic leadership emergence. While the article was informative, it did not meet our aim.
• Webster et al. [5] conducted primary qualitative and quantitative research into the common responses of followers coping with toxic leadership. Their study had a sample size of 74, but since only 14 health professionals were included, it is not possible to draw conclusions from this article about the extent to which toxic leadership impacted the psychological well‐being of these professionals.

A citation search was conducted by reviewing the reference list of the seven articles to identify any related literature that could be included in the review. Additionally, a Google Scholar citation search was conducted to identify relevant studies that cited the seven articles. This resulted in the inclusion of three additional articles in this study.

Guided by the PRISMA extension for scoping reviews (PRISMA‐ScR), all three authors conducted a manual thematic analysis to identify themes and patterns based on our interpretations of findings and recommendations of the included articles. Each author independently reviewed the articles, highlighting relevant points of interest that aligned with the research question. These points were subsequently discussed among all the authors, and agreed‐upon themes were identified. To ensure consistency, a comparative table outlining the key themes was created and critically reviewed by all authors to accept and reconcile any discrepancies. This iterative process ensured that all opinions and thoughts were considered, and the final themes accurately reflected the findings across the studies. According to Hong et al. [24], appraising the quality of primary studies is a core step in systematic reviews and involves a careful examination to ensure the review is trustworthy, valid, and reliable. Given the lack of qualitative and mixed‐methods articles that resulted from our systematic search, it was only possible to rely on evidence from quantitative research.

**FIGURE 1:**
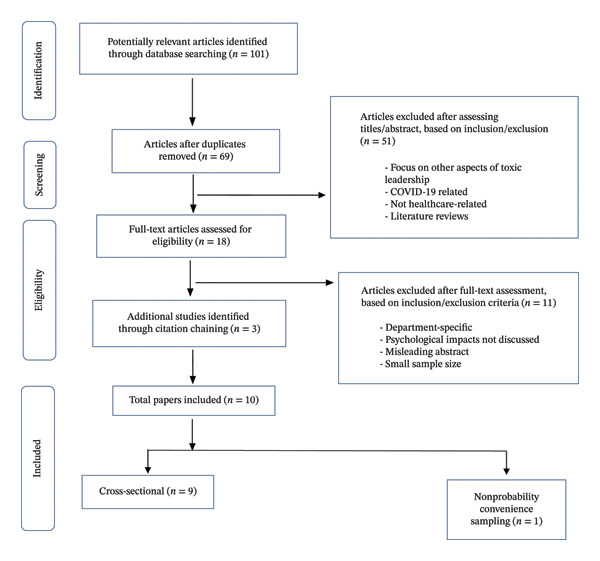
Modified PRISMA ScR framework flow diagram of article screening and selection.

Table 1 presents the databases and search terms used to identify relevant literature for review (the table was adapted from Brownie and Nancarrow [25]). Figure 1 illustrates the PRISMA framework flow diagram (the flow diagram was modified from Brownie and Nancarrow [25]).

## 3. Results

In total, 10 international articles met the inclusion criteria and were used in this review, as presented in Table 2. The studies consisted of one nonprobability convenience sampling [26] and nine cross‐sectional [7, 10, 11, 27–32]. The studies’ countries of origin were broad, comprising Canada (*n* = 2), Pakistan (*n* = 2), and one each from China, Egypt, France, Germany, the Philippines and Turkey.

**TABLE 2:** Key methodological features and quantitative findings from reviewed studies.

Author, (year), country	Aim(s)	Study design	Sample/participants	Data collection and analysis	Key findings/outcome (s) statistically significant *p* ≤ 0.05 (two‐sided hypothesis), *p* ≤ 0.01 (one‐sided hypothesis), highly significant *p* ≤ 0.001	Limitations
Ahmed et al. [27] Egypt	Investigate the correlation between toxic leadership and workplace deviance.	Descriptive Cross‐sectional	Participants: = 243 Gender: Female: *n* = 169 (70%) Male: *n* = 74 (30%) Occupation: Nurses Multicentre = 3 university hospitals	4 questionnaires: Tox‐BH‐NM 9‐item Emotional Exhaustion Scale 25‐item Workplace Deviance Scale 16‐item Organisational Cynicism Scale Analysis: AMOS 24, SPSS 29 and Pearson correlation	Toxic leadership significantly and positively correlated with emotional exhaustion (*p* < 0.001). Higher levels of toxic leadership are closely linked with increased workplace deviance, emotional exhaustion and organisational cynicism, highlighting the negative effects on both the individual and the organisation.	Small sample size Questionnaire‐based with no direct observation Consider a qualitative or mixed‐method approach in future

Chaudhary and Islam [28]. Pakistan	How does despotic leadership affect employees’ psychological distress?	Cross‐sectional	Participants: *n* = 252 Gender: Female: *n* = 205 (81%) Male: *n* = 47 (19%) Occupation: Nurses and supervisors Multicentre = 7 public and private hospitals	4 questionnaires: 6‐item Despotic Leadership 6‐item Bullying behaviour 6‐item Hostile Attribution Bias 6‐item Psychological Distress Analysis: SEM	Despotic leaders can trigger employees’ bullying behaviour, which further affects psychological distress (*p* < 0.002). Despotic behaviours negatively affect employee health, including psychological distress.	Consider a longitudinal approach for future research to mitigate common method bias

Durrah et al. [10] France	How toxic management styles can lead to both psychological and physical withdrawal.	Quantitative	Participants: *n* = 413 Gender: Female: *n* = 172 (42%) Male: *n* = 241 (58%) Occupation: Hospital managers, frontline department heads, nurses, assistants and healthcare technicians Multicentre = 2 public and 2 private hospitals	2 questionnaires: 13‐item Toxic leadership 11‐item Withdrawal behaviours Analysis: Smart PLS 3.3.9 program	Supervisors who self‐promote are the most impactful for psychological withdrawal (*p* < 0.002). A supervisor’s unpredictable behaviours positively and significantly relate to psychological withdrawal (*p* < 0.001). There is a significant correlation between supervisors who self‐promote and psychological withdrawal.	Consider a qualitative approach to obtain in‐depth results on psychological impacts

Erschens et al. [29] Germany	Association of general well‐being and different leadership styles among employees in a German hospital.	Cross‐sectional	Participants: *n* = 1137 Gender: Female: *n* = 850 (75%) Male: *n* = 287 (25%) Occupation: Administration, Physicians, nurses and others. One site hospital	2 questionnaires: 36‐item Module A and D from the Integrative Leadership questionnaire 5‐item World Health Organisation Well‐being Index (WHO‐5) Analysis: *T*‐test and ANOVA with IBM SPSS 25	Laissez‐faire (LF) and destructive leadership (D) were negatively correlated with the WHO‐5 “Well‐being” questionnaire, with *r* = −0.24 (LF) and *r* = −0.26 (D) (two‐tailed α = 0.001). Contrarily, it was found that transformational leadership had a positive and significant relationship with employees’ well‐being (*p* < 0.001). Leadership behaviour is associated with the well‐being of those being led.	Consider a longitudinal approach for future research.

Koç et al. [7] Turkey	The effect of toxic leadership on emotional exhaustion in healthcare.	Cross‐sectional	Participants: *n* = 133 Gender: Female: *n* = 122 (92%) Male: *n* = 11 (8%) Occupation: Nurses Age: 28–35 years Multicentre = public and private hospitals, with the number of sites unknown	3 questionnaires: Tox‐BH‐NM 9‐item Emotional Exhaustion Scale 6‐item Intrinsic Motivation Scale Analysis: PLS‐SEM technique	Toxic leadership is positively and significantly associated with emotional exhaustion (*p* < 0.01). Toxic leadership is an antecedent of emotional exhaustion.	Small sample size Younger cohort of participants

Labrague et al. [30] Philippines	Influence of toxic and transformational leadership on nurses’ job satisfaction, psychological distress, absenteeism and intent to leave the organisation.	Cross‐sectional	Participants *n* = 770 Gender: Female: *n* = 451 (59%) Male: *n* = 319 (41%) Occupation: Nurses Age: mean 28.5 years Multicentre: 15 public and private hospitals	4 questionnaires: Tox‐BH‐NM 7‐item Global Transformational Leadership (GLT) Scale 16‐item Job Satisfaction Index 4‐item Perceived Stress Scale Analysis: SPSS 22 and Pearson correlation	Toxic leadership correlated significantly with psychological distress (*p* < 0.05). Toxic leadership is associated with increased psychological distress	Consider a qualitative approach to assist with context

Lyu et al. [31] China	Determine the mediating effects of psychological empowerment on abusive supervision	Descriptive Cross‐sectional	Participants: *n* = 1127 Gender: Female: 1066 (95%) Male: *n* = 61 (5%) Occupation: Nurses Multicentre: 4 public hospitals	3 questionnaires: 15‐item Abusive supervision 12‐item Psychological empowerment 5‐item Turnover intention Analysis: IBM SPSS 19, Pearson correlation and regression analysis	Perceived abusive supervision and psychological empowerment were significantly negatively correlated (*p* < 0.001) Psychological empowerment mediates abusive supervision.	A longitudinal multicentre study could be considered

Majeed and Fatima [26]. Pakistan	Impact of exploitative leadership on psychological distress and the moderating role of psychological detachment from work between exploitative leadership and negative affectivity.	Nonprobability convenience sampling Three‐time lag of 15 days	Participants: *n* = 231 Gender: Female: *n* = 206 (89%) Male: *n* = 25 (11%) Occupation: Nurses Multicentre: 15 hospitals	4 questionnaires: 15‐item Exploitative leadership 10‐item Negative affectivity 10‐item Psychological distress 4‐item Psychological detachment Analysis: SPSS 20 with F‐test one‐way ANOVA	Direct exploitative leadership is significantly and positively related to psychological distress (*p* < 0.001) and negative affectivity (*p* < 0.001). Negative affectivity is significantly and positively related to psychological distress (*p* < 0.001). Exploitative leadership can lead to psychological distress Negative affectivity mediates the relationship between exploitative leadership and psychological distress	Using a longitudinal approach rather than a time‐lagged research design is a consideration for future studies to determine if negative outcomes persist over time.

Mullen et al. [32] Canada	Examine the relationship between abusive supervision and employee health and safety.	Cross‐sectional	Participants: *n* = 145 Gender: Female: *n* = 123 (85%) Male: *n* = 22 (15%) Occupation: Nurses, social workers and administration Age: mean 43.47 years Multicentre without specific detail	4 questionnaires: 15‐item Abusive supervision 10‐item Safety climate 4‐item Safety participation 12‐item Psychological health Analysis: Maximum likelihood estimation	Abusive supervision predicted psychological health (*p* < 0.01). Abusive supervision has a negative impact on employee psychological health. Uncivil behaviours are harmful to employees.	Small sample size. Consider longitudinal or experimental approaches for future research.

Trépanier et al. [11] Canada	Investigate the psychological and motivational processes involved in the relationship between tyrannical and laissez‐faire leadership and health.	Cross‐sectional	Participants: *n* = 399 Gender: Female: *n* = 351 (88%) Male: *n* = 48 (12%) Occupation: Nurses Age: mean 42.74 years Details about the hospital and location were not provided.	6 questionnaires: 4‐item Destructive Leadership Scale 9‐item Psychological Need Thwarting Scale. 8‐item Two modules from The Multidimensional Work Motivation Scale. 10‐item Maslach Burnout Inventory General Survey 6‐item Occupational Commitment A self‐reported job performance scale Analysis: SEM	Controlled motivation as a trait of tyrannical leadership (68%) as well as laissez‐faire leadership as a whole (20%) was positively associated with burnout (*p* < 0.01). Destructive leadership is associated with various symptoms of promoting negative psychological experiences.	Consider longitudinal research for future research

Hospital nurses were the primary cohort of participants in the studies (*n* = 6). This is expected, as nurses worldwide constitute the largest workforce within the healthcare sector [33]. Chaudhary and Islam [28], Durrah et al. [10], Erschens et al. [29] and Mullen et al. [32] conducted studies on healthcare workers (*n* = 4), comprising hospital nurses, care assistants, health managers, social workers, physicians, administrators and technicians.

All 10 studies utilised questionnaires and were of a quantitative nature. Aside from the one mixed‐method study by Webster et al. [5], the search yielded no other qualitative or mixed‐method studies. In total, across the 10 studies, 4850 participants completed questionnaires, comprising 3715 females (77%) and 1135 males (23%). Durrah et al. [10] had the highest number of male participants, with 241 (57%).

The smallest study, conducted by Koç et al. [7], involved 133 participants, while the largest, from Erschens et al. [29], comprised 1137 participants. Erschens et al. [29] was the only study performed using a single‐site hospital. Trépanier et al. [11] did not specify the location; the remaining studies were conducted at multiple sites, including public, private and university hospitals.

The studies employed various closed questionnaires and utilised Likert scales for responses. Across the 10 studies, 33 questionnaires were used, ranging between two and six questionnaires per study, with Durrah et al. [10] and Erschens et al. [29] asking the fewest questions and Trépanier et al. [11] asking the most.

Majeed and Fatima [26] conducted their study with three‐time lags of 15 days each to mitigate common method bias. Labrague et al. [34] developed the most widely used questionnaire in the studies included in our scoping review, the Toxic Leadership Behaviours of Nurse Managers (Tox‐BH‐NM) Scale, to measure nurses’ experience of toxic leadership. The scale of the Tox‐BH‐NM comprises 30 questions across four behaviours: humiliating (3), intemperate (15), narcissistic (9) and self‐promoting (3) [27]. Ahmed et al. [27], Koç et al. [7] and Labrague et al. [30] utilised the Tox‐BH‐NM as one of their assessment tools.

Nine of the 10 studies investigated the direct links between toxic, abusive, destructive and despotic leadership styles and the psychological impacts on professionals due to this behaviour. Erschens et al. [29] investigated the association between perceived leadership style and the subjective well‐being of employees, which included the psychological impacts on employees. Lyu et al. [31] investigated the mediating effects of psychological empowerment on abusive supervision and turnover intention, which also highlighted the psychological impacts of toxic behaviour on employees.

Five key themes were identified across the 10 studies. (1) The first theme confirmed that toxic leadership can cause adverse psychological impacts on professionals. There was a significant correlation between toxic leadership and direct psychological impacts on staff, described by Chaudhary and Islam [28], Durrah et al. [10], Labrague et al. [30], Lyu et al. [31], Majeed and Fatima [26], Mullen et al. [32] and Trépanier et al. [11]. (2) The second theme highlighted the need to train and develop managers in toxic leadership and behaviours, and their negative impacts on employees [7, 10, 11, 26, 27]. (3) The third theme, as highlighted in four studies, is the benefits of the transformational leadership style versus toxic leadership [10, 29, 30, 32]. Erschens et al. [29] specifically discuss that transformational and transactional leadership styles increase staff well‐being. (4) The fourth theme, identified by Koç et al. [7], Mullen et al. [32] and Trépanier et al. [11], suggests that further research is needed into the potential costs associated with toxic leadership in an already under‐resourced healthcare environment. This could include investigating staff absenteeism and turnover rates, as well as increases in workers’ compensation premiums and recruitment costs, resulting from toxic leadership. (5) The fifth theme was identified by Ahmed et al. [27] and Chaudhary and Islam [28] and revolves around the benefits of 360‐degree appraisals in providing feedback and supporting professional development to assist in reducing toxic leadership. This approach can be adopted by all employees in supervisory positions.

All articles supported that staff exposed to toxic leadership can experience adverse psychological impacts. The findings confirm that psychological impacts for professionals encountering toxic leadership include anxiety, burnout, depression, emotional exhaustion, low motivation, malaise, negative affectivity, stress and withdrawal [10, 26, 28, 29, 31, 32]. A concerning consequence of toxic leadership is that bullying behaviours can also be triggered by professionals who experience this style of leadership, which in turn spreads the toxicity to others [28].

Table 2 presents the key methodological features of the reviewed studies.

## 4. Discussion

This scoping review aimed to explore research examining the psychological impacts of toxic leadership within the healthcare sector in general and hospital nursing specifically. The impacts of toxic leadership behaviours can be dehumanising and demotivating for professionals, resulting in feelings of reduced confidence, disempowerment, self‐doubt, mistrust, anger and vulnerability [35]. Our analysis revealed that toxic leadership in healthcare settings poses significant risks to professionals’ psychological and physical well‐being and is a growing worldwide concern. It not only leads to significant organisational costs, such as increased absenteeism, turnover, compensation claims and reputational damage, but can also perpetuate harmful behaviours, with affected staff sometimes exhibiting bullying toward subordinates. In contrast, half of the analysed studies support that transformational leadership in recent years has emerged as the gold standard, offering a proven path to fostering positive workplace culture and improving outcomes across the board. Hansen and Pihl‐Thingvad [36] describe transformational leadership as one of the positive leadership styles in which leaders use their influence to develop a vision by inspiring innovation, communicating effectively and motivating employees to achieve organisational goals.

The key findings reinforce the theoretical typologies of toxic leadership. It supports the consequences of toxic leadership and the resulting adverse psychological impacts on professionals, which were not unexpected findings. For example, Labrague et al. [30] demonstrated that toxic leadership arises from the interaction between a toxic leader, a follower, and a conducive environment, referred to as the toxic triangle. A few years earlier, Fraher [37] called this psychological construct “organisational Munchausen syndrome by proxy”, revealing how destructive leaders and susceptible followers can be drawn into dysfunctional dynamics that can escalate into disaster or death, as Fraher believes might have been the case in the paediatric cardiac deaths at the Bristol Royal Infirmary in the United Kingdom in the late 1980s and early 1990s.

It is vital to remember the impacts toxic leadership can have within the healthcare sector, where patients’ lives are at stake, compared to other industries. Within the healthcare sector, toxic leadership can have a profoundly negative impact on patient care, safety and outcomes [10]. It was most concerning to learn that toxic leadership can trigger bullying behaviours in affected professionals. It would be assumed that if a person were being treated poorly, they would not exhibit these behaviours towards others.

## 5. Strengths and Limitations

A major strength of this scoping review was the validation of the theoretical literature on toxic leadership and its psychological impacts on health professionals, particularly hospital nurses, worldwide, thereby enabling the research question to be answered. The period from 2014 to 2025 was a strength in maintaining the currency of evidence. Another strength was that the 10 quantitative studies used multiple closed‐ended questionnaires across a total of eight countries, resulting in robust evidence. The quality of the studies was high (as per the Critical Appraisal Skills Programme [CASP] guidelines), with all articles providing informative summaries of critical appraisal to demonstrate the validity of the research, correlations, variances, data collection, analysis and quality.

It was remarkable that no qualitative or mixed‐method studies met the inclusion criteria, at least not in the peer‐reviewed literature, which is noted as a limitation. Given the topic and the research question, an assumption was first made that more qualitative studies would have been available. The primary research available relies solely on quantitative questionnaires, without integrating observations and verbal communication, which could introduce bias and fail to capture the intricacies of respondents’ experiences and workplace environments and restricts the level of understanding of the psychological experiences of toxic leadership. To gain an in‐depth understanding of how hospital nurses experience toxic leadership, qualitative research is required. Had we also included grey literature in our review, we might have found PhD dissertations and reports written by respected research institutes, as well as unpublished qualitative research or quality improvement projects, that could have provided a better understanding of how toxic leadership is experienced.

This scoping review comprised 4850 responses, with the majority of respondents being nurses. Nine articles identified 3499 nurses (this was not clearly identified in the tenth article), representing 70% of the total respondents. The vast majority of nurses in the complete study population is both a strength and a limitation; as we initially wanted to focus on health professionals in general, a limitation of our review is the sample size (*n* = 1401; 30%) from non‐nursing professions, which are not comprehensively represented. Future studies should ensure proportional representation.

The inconsistent application of pre‐ and postdata collection is a methodological limitation as well as the reliance on self‐reported questionnaires which imply the risk of self‐bias. Koo and Yang [38] discuss the implications of response bias, in which participants’ responses may be influenced by external factors rather than their own beliefs or experiences, leading to skewed data. This can affect the reliability of the analysed studies and consequently of this scoping review. The dominance of cross‐sectional studies did not allow for determining causation or the long‐term consequences of toxic leadership.

It should also be noted as a limitation that only articles printed in the English language were used as part of this review; more articles could be available in other languages.

## 6. Implications

This review has highlighted the concern that there is significant evidence confirming that one of the many adverse effects of toxic leadership is the psychological impact caused within healthcare. The evidence supports the need for change within healthcare to manage toxic leadership and behaviours. All the articles provided rational recommendations for managing toxic leadership and its subsequent outcomes, ranging from implementing leadership training for managers and overhauling recruitment processes to providing counselling for employees, fostering positive workplace environments and investigating associated costs.

Reflecting on the findings and recommendations from the 10 articles, the following recommendations for both public and private healthcare providers should be considered. First (1), Hospital Administration, Executive and Boards need to be informed about the multiple impacts of toxic leadership on their organisation, employees, patients and communities. It is essential that they are all committed to eradicating toxic leadership by actively recognising the issue, being accountable, introducing change, addressing concerns, promoting transformational leadership and supporting a positive workplace culture. Second (2), recruitment processes for employees in supervisory positions should include an assessment tool to detect toxic behaviours as part of the prescreen or in the interview process. The reference request for prospective employees could also include a question about toxic leadership or leadership styles. Third (3), leadership training should be provided for all leaders (regardless of seniority), explicitly addressing the impacts that toxic leadership can have but also how self‐awareness and transformational leadership can be adequately utilised. In here, a 360‐degree appraisal should be implemented, enhanced and adopted by all leaders. Finally (4), organisations should actively foster and promote a positive workplace culture by ensuring that there is adequate support for employees who have been affected by toxic leadership, including counselling, coping techniques and mentoring.

Further research could investigate whether there is a relationship between toxic leadership and poor safety outcomes, as well as the outcomes for staff who have been adversely affected. Additionally, the opinions of the Administration, Executive, and Board could be explored regarding their experiences and strategies for eradicating toxic leadership, as well as for rebuilding, transforming and transitioning to a culture of transformational leadership. Quantitative research with a control group could potentially demonstrate causal relationships. However, the subject of toxic leadership does not lend itself to randomised controlled trials because this can involve various ethical dilemmas. Therefore, forms of qualitative research such as in‐depth qualitative case‐studies with a small group of participants who are (or were) victims of toxic leadership, can possibly reveal causal relationships. Further, participatory action research can be considered, where both staff and management can learn from each other’s experiences. Finally, the associated costs of toxic leadership could be explored to estimate the costs of this leadership style.

## 7. Conclusion

The aim of this review was to identify the psychological impacts on health professionals, in general, and on hospital nurses specifically, resulting from toxic leadership by managers. Commonly seen psychological impacts were identified as anxiety, burnout, depression, emotional exhaustion, low motivation, malaise, negative affectivity, stress and withdrawal, all experienced worldwide.

Combined, the studies and review provide a significant contribution to the nursing leadership literature by evidencing the various psychological impacts on hospital nurses. In addition, other consequences of toxic leadership were also highlighted, including the physical impacts on staff.

It is recommended that organisations adopt and support the transformational leadership style, coupled with 360° appraisals, which, for some organisations, will require an overhaul and transition from current business practices. Future research into the experiences of health professionals under toxic leadership and culture, as well as research into its associated costs, could provide a more in‐depth understanding of its psychological impacts on staff and the financial consequences for organisations. Conducting this research will provide nursing management and policymakers with the confidence to implement new processes for managing this dark side of leadership.

When healthcare organisations start taking accountability and implement changes to eliminate toxic leadership styles, this will, in turn, reduce negative impacts on staff, increase retention, improve financial performance and service delivery, and foster a positive work culture. This ultimately creates an improved environment in which high‐quality and safe care can be provided to our patients.

## Author Contributions

Conceptualisation: Alix Cooke and Gideon de Jong. Data curation: Alix Cooke. Formal analysis: all authors. Funding acquisition: N/A. Investigation: Alix Cooke. Methodology: all authors. Project administration: Alix Cooke and Gideon de Jong. Resources: Alix Cooke and Gideon de Jong. Software: N/A. Supervision: Gideon de Jong. Validation: all authors. Visualisation: Alix Cooke. Writing–original draft preparation: Alix Cooke. Writing–review and editing: all authors.

## Funding

No funding was received. Open access publishing facilitated by Southern Cross University, as part of the Wiley ‐ Southern Cross University agreement via the Council of Australasian University Librarians.

## Conflicts of Interest

The authors declare no conflicts of interest.

## Data Availability

Data are available on request from the authors.
